# Roentgen Rays in Dentistry

**Published:** 1901-09-15

**Authors:** W. A. V. Price


### ROENTGEN RAYS IN DENTISTRY.

BY W. A. V. PRICE, D.D.S.

Synoptic report of a lecture before Tri-State Dental Meeting, Indianapolis, June, 1901.

Dr. Price gave a series of lantern pictures illustrating the application of the X-ray in diagnostic work in dentistry. The first set of pictures showed value of a skiagraph picture in locating undeveloped teeth and other abnormal conditions which could not possibly be discovered without extensive operations. The different tissues transmit the rays differently. The gum tissue does not produce a shadow on the photographic plate. Pus deposits are readily distinguished from soft tissues which surround them. Tooth and bone tissues are very clearly outlined in deep shadows, so that their form and size are clearly seen. Imperfect root fillings are distinguishable, also imperfections about fillings of all kinds. Certain diseased conditions of the antrum are readily made out, and the relations of alveolar abscesses to antral or nasal troubles are clearly made out.

Dr. Price has succeeded in simplifying the methods of taking the pictures so that but a few seconds suffice to enable him to produce very fine pictures. The apparatus is somewhat expensive. It costs about two hundred and fifty dollars to put in an apparatus which will prove effective.

Dr. Price made as a practical demonstration several pictures of a boy who had never erupted the laterals or second bicuspids. The pictures were developed and showed no signs of the missing teeth, and the probabilities are that he will never erupt any of them. The pictures and apparatus, with the facility with which it can be used, was a convincing argument for its adoption in dental practice. Dr. Price also called attention to the use of the ray as a therapeutic remedy of considerable value.

For dental purposes the static machine is not used because of its bulky character. The induction coil with a Wehnelt interrupter as made by Dr. Price, with special tubes, gives the best and quickest results. The pictures are made on a specially prepared film, which is held in sheets of rubber and can be cut to any desired form or size. The systematic manner in which this subject has been worked up for dental purposes, leaves little to be desired, and the profession is to be congratulated for having made so valuable an addition to its remedial resources.

